# Genetic Predisposition to Higher Body Mass Index or Type 2 Diabetes and Leukocyte Telomere Length in the Nurses' Health Study

**DOI:** 10.1371/journal.pone.0052240

**Published:** 2013-02-12

**Authors:** Mengmeng Du, Jennifer Prescott, Marilyn C. Cornelis, Susan E. Hankinson, Edward Giovannucci, Peter Kraft, Immaculata De Vivo

**Affiliations:** 1 Department of Epidemiology, University of Washington School of Public Health, Seattle, Washington, United States of America; 2 Public Health Sciences Division, Fred Hutchinson Cancer Research Center, Seattle, Washington, United States of America; 3 Channing Laboratory, Department of Medicine, Brigham and Women's Hospital and Harvard Medical School, Boston, Massachusetts, United States of America; 4 Program in Molecular and Genetic Epidemiology, Harvard School of Public Health, Boston, Massachusetts, United States of America; 5 Department of Nutrition, Harvard School of Public Health, Boston, Massachusetts, United States of America; 6 Division of Biostatistics and Epidemiology, University of Massachusetts School of Public Health and Health Sciences, Amherst, Massachusetts, United States of America; 7 Department of Epidemiology, Harvard School of Public Health, Boston, Massachusetts, United States of America; Peninsula College of Medicine and Dentistry, United Kingdom

## Abstract

**Background:**

Although cross-sectional studies have linked higher body mass index (BMI) and type 2 diabetes (T2D) to shortened telomeres, whether these metabolic conditions play a causal role in telomere biology is unknown. We therefore examined whether genetic predisposition to higher BMI or T2D was associated with shortened leukocyte telomere length (LTL).

**Methodology:**

We conducted an analysis of 3,968 women of European ancestry aged 43–70 years from the Nurses' Health Study, who were selected as cases or controls in genome-wide association studies and studies of telomeres and disease. Pre-diagnostic relative telomere length in peripheral blood leukocytes, collected in 1989–1990, was measured by quantitative PCR. We combined information from multiple risk variants by calculating genetic risk scores based on 32 polymorphisms near 32 loci for BMI, and 36 polymorphisms near 35 loci for T2D.

**Findings:**

After adjustment for age and case-control status, there was no association between the BMI genetic risk score and LTL (β per standard deviation increase: −0.01; SE: 0.02; *P* = 0.52). Similarly, the T2D genetic score was not associated with LTL (β per standard deviation increase: −0.006; SE: 0.02; *P* = 0.69).

**Conclusions:**

In this population of middle-aged and older women of European ancestry, those genetically predisposed to higher BMI or T2D did not possess shortened telomeres. Although we cannot exclude weak or modest effects, our findings do not support a causal relation of strong magnitude between these metabolic conditions and telomere dynamics.

## Introduction

Telomeres are repetitive DNA-protein complexes that protect the ends of linear chromosomes and maintain genomic stability [1]. They shorten with age, and biological factors such as oxidative stress and inflammatory responses accelerate leukocyte telomere length (LTL) attrition [2], [3]. Thus, LTL may reflect cumulative exposure to oxidative and inflammatory damage, serving as a potential indicator of cellular aging [2]. Given that shortened telomeres may be associated with increased incidence and mortality of many age-related diseases [3], [4], [5], [6], [7], [8], [9], it is important to better understand factors that influence telomere biology.

Like telomere length, obesity and type 2 diabetes (T2D) have been associated with oxidative stress, inflammation, and various age-related outcomes [10], [11], [12], [13]. However, whether these metabolic conditions are associated with telomere length, as well as the direction of these relations, are unclear. Studies have shown associations between higher body mass index (BMI) [14], [15], [16], [17] or T2D [12], [18], [19], [20] and telomere shortening, although some have reported no relation [21], [22], [23]. While these associations may be explained by heightened oxidative stress and inflammation among obese or diabetic individuals, which promote telomere erosion, recent findings have suggested telomeres may play a role in the development of obesity or T2D [24], [25]. The paucity of prospective epidemiologic data, however, precludes the evaluation of these conditions as a cause or consequence of shortened telomeres. Moreover, as many studies reported unadjusted or age-adjusted results, it is also unclear whether observed associations were influenced by unmeasured confounders such as smoking or physical activity.

To address these questions, we examined whether genetic predisposition to higher BMI or T2D was associated with shortened LTL among 3,968 women of European ancestry aged 43–70 years. Because each individual risk variant confers modest risk, we combined genotype information across 32 established risk variants near 32 loci for higher BMI [26], or 36 established risk variants near 35 loci for T2D [27], to provide a global measure of genetic predisposition. As genotypes are unaffected by telomere shortening and independent of most confounders [28], [29], we used these genetic risk scores as proxies for higher BMI and T2D exposure to evaluate whether these metabolic conditions play a causal role in LTL dynamics.

## Materials and Methods

### Ethics statement

Completion of the self-administered questionnaire and submission of a blood sample were considered to imply informed consent. The protocol for this study was approved by the Human Research Committees at Brigham and Women's Hospital, Boston, MA, USA (IRB protocol number: 1999-P-010363).

### Study population

The Nurses' Health Study (NHS) prospective cohort was established in 1976, when 121,700 female registered nurses aged 30 to 55 years completed and returned a mailed questionnaire. Information on individual characteristics and new disease diagnoses has been updated biennially. From 1989 to 1990, 32,826 women provided blood samples and completed a questionnaire. Details of the NHS and blood collection methods have been described previously [30].

Among women who had previously provided a blood sample, individuals of European ancestry were included as cases or controls in genome-wide association studies of incident cancers, cardiovascular disease, T2D, kidney stones, or open-angle glaucoma [31], [32], [33], [34]. Also among women who had previously provided a blood sample, individuals were selected to participate in nested case-control studies of LTL and incident cancers or cardiovascular disease [35], [36], [37]. In studies of LTL, eligible cases were identified starting any time *after* blood collection in 1989–1990 (i.e., after telomere length assessment) up to June 1, 2008. Participants included in the present analysis comprised those selected in both genome-wide association studies *and* nested case-control studies of telomeres and disease. The present analysis excluded women with extreme LTL values (*N* = 14) or LTL values generated by non-standard assay conditions (*N* = 218). The final study population included 3,968 women of European ancestry.

### Assessment of covariates

From the questionnaire completed at blood collection, we assessed age, BMI (calculated as weight in kilograms/height in meters^2^), and date of blood collection. From questionnaires administered before or at approximately the time of blood collection, we assessed various anthropometric, reproductive, and lifestyle factors, as well as the presence or history of chronic diseases, including T2D status. We defined T2D as initially self-reported diabetes subsequently confirmed by a validated supplementary questionnaire [38], [39], [40]. T2D before 1998 was diagnosed using the National Diabetes Data Group criteria [40], [41]. T2D after 1998 was diagnosed using the American Diabetes Association criteria [42]. Using a semi-quantitative food frequency questionnaire [43] administered in 1990, we assessed intakes of dietary factors. Because the genetic score may best reflect long-term BMI, we calculated a cumulative average measure by averaging BMI data from all available questionnaires up to the time of blood collection.

### Measurement of relative leukocyte telomere length

Genomic DNA was extracted from peripheral blood leukocytes using the QiAmp (Qiagen, Valencia, CA, USA) blood kit. We assessed relative LTL using quantitative polymerase chain reaction [44]. Average relative LTL was calculated as the exponentiated ratio of Telomere repeat copy number to Single gene (*36B4*) copy number (T/S) [45]. Laboratory technicians masked to participant characteristics assayed each sample in triplicate. Quality control samples were interspersed on each plate to assess variability. In all nested case-control studies of LTL, coefficients of variation (CVs) for the telomere and single gene assay were less than 4%, and CVs for the exponentiated T/S ratio were less than 18%. Although this assay provides a relative measurement of telomere length, T/S ratios correlate well with absolute telomere lengths determined by Southern blot (*r* = 0.82, *p*<0.0001) [45].

### Genotyping

Detailed information on genotyping and quality control procedures has been described elsewhere [31], [32], [33], [34]. Briefly, DNA samples in various genome-wide studies were genotyped using either Affymetrix 6.0 (Affymetrix, Santa Clara, CA, USA) or Illumina arrays (HumanHap550k, 610Q, 660W, OmniExpress) (Illumina, Inc., San Diego, CA, USA). After genotyping, quality control filters were applied: We removed single nucleotide polymorphisms (SNPs) with low minor allele frequencies, low call rates, or genotype frequencies that significantly departed from Hardy-Weinberg equilibrium, as well as individuals with outlying missing genotype or heterozygosity rates, or those of non-European ancestry as determined by principal components analysis [46]. MACH [47] was used to impute autosomal SNPs using NCBI build 36 of Phase II HapMap CEU data (release 22) as the reference panel.

### Calculation of genetic risk scores

To reflect genetic predisposition to higher BMI, we selected 32 independent polymorphisms near 32 loci recently established in a meta-analysis of genome-wide association studies that included ∼250,000 individuals [26] (Table S1 in File S1). For genetic predisposition to T2D, we selected 36 independent polymorphisms near 35 loci that have been robustly replicated in genome-wide association studies of T2D [27] (Table S2 in File S1). Most BMI or T2D risk variants were genotyped or had high imputation quality (MACH ‘Rsq’≥0.8). In sensitivity analyses, results were essentially unchanged after excluding SNPs imputed with MACH Rsq<0.8 (3 BMI SNPs; 6 T2D SNPs).

To maximize the strength of the genetic scores as proxies for BMI and T2D, we calculated the scores using a weighted method that accounted for the strength of prior associations. We multiplied the number of risk alleles (i.e., allele associated with higher BMI or risk of T2D) for each SNP by its previously published relative effect size (β-coefficient) before summing the products. For interpretability, the weighting process created a score for which each unit corresponded to an average risk allele. Detailed calculations for the weighted genetic scores have been described previously [26], [38]. Risk variants in the scores were chosen specifically based on their established effects on BMI or T2D, and we assumed these scores were not associated with telomere length through factors or mechanisms unrelated to these conditions [28], [29]. Few participants had incomplete genotype data (*N* missing 1 or 2 SNPs: BMI, 82; T2D, 45). We assigned missing genotypes the average genotype at that locus. In sensitivity analyses, exclusion of individuals with missing genotypes did not alter the findings.

### Statistical analyses

We calculated the natural logarithm of LTL to better approximate normality. Among participants in each nested case-control set, we identified outlying LTL values using the generalized extreme studentized deviate many-outlier procedure [48]. To ensure comparability across sets for relative LTL values, we computed *z*-scores of LTL for individuals in each set using the formula *z-score*  =  (*x*−*μ*)/*σ*, where *x* is an individual's LTL, *μ* is the set-specific mean LTL, and *σ* is the set-specific standard deviation of LTL.

To analyze additive associations (0, 1, or 2 copies of the risk allele) between each BMI- or T2D-related variant and LTL, we used linear regression adjusted for age and case status. We conducted exact binomial tests to assess whether more risk variants than expected by chance were significantly associated with shorter telomeres. In addition, we performed False Discovery Rate (FDR) corrections to account for multiple statistical tests [49]. To evaluate the suitability of the genetic score as a proxy for BMI, we estimated least squares mean BMI using generalized linear models. For T2D, we estimated odds ratios and 95% CIs using unconditional logistic regression.

To examine associations between the genetic scores and LTL, we estimated adjusted least squares mean LTL (*z*-scores) by genetic score quartiles using generalized linear models adjusted for age and case status. We also considered the following factors potentially associated with LTL: date of blood collection, nested case-control study of LTL, genome-wide association study, pack-years of smoking, menopausal status, past or current postmenopausal hormone therapy, physical activity, paternal age, and family history of diabetes. As results were similar after including these variables, they were omitted from the primary models. We tested for linear trend by separately including each genetic score as a continuous predictor in multivariable models.

Because the genetic effects of BMI- or T2D-related variants may vary by lifestyle [50], [51], we conducted exploratory analyses to evaluate whether associations differed by unhealthy lifestyle pattern expected to contribute to weight gain, calculated by creating low- or high-risk variables using the median value of four lifestyle factors: physical activity, sitting, sweetened beverage intake, alternate healthy eating index [52], and then summing the number of high-risk variables. We used partial *F*-tests to compare nested models with and without interaction terms between the genetic scores and lifestyle variable.

*P*-values were 2-sided and *P*≤0.05 was considered significant. All statistical analyses used SAS, Version 9.2, software (SAS Institute Inc, Cary, NC) or R, Version 2.11.1 (R Foundation for Statistical Computing, Vienna, Austria).

## Results

The mean age of participants was 59.2 years. As expected, women with longer telomeres were younger, less likely to be obese, and less likely to have T2D compared with women with shorter telomeres (Table 1). After adjustment for potential predictors of telomere length (e.g., age, case status, smoking, physical activity, menopausal status, postmenopausal hormone therapy), LTL was inversely associated with age (−0.065 SD per 5-yr increase; SE: 0.014; *P*<0.001), BMI (−0.043 SD per 5 kg/m^2^ increase; SE: 0.020; *P* = 0.031), and T2D (−0.116 SD for diabetics vs non-diabetics; SE: 0.060; *P* = 0.055). In addition, women with longer telomeres smoked fewer pack-years, tended to be more active, and were less likely to currently use postmenopausal hormone therapy (Table 1).

**Table 1:** Age and age-adjusted characteristics of 3,968 women in the Nurses' Health Study by telomere length (*z*-score), 1989–1990.a,b

	Quintile of LTL (*z*-score)		
Characteristic	5^th^ (longest)	3^rd^	1^st^ (shortest)
*N*	790	791	795
LTL (*z*-score)	1.3 (0.4)	−0.01 (0.1)	−1.4 (0.5)
Age at blood collection (yrs)^c^	58.8 (6.7)	58.9 (6.3)	60.0 (6.4)
Body mass index (kg/m^2^)			
18.5–<25	51.1%	49.5%	50.5%
25–<30	31.3%	31.6%	30.0%
≥30	16.5%	18.0%	18.2%
Type 2 diabetes	6.9%	7.3%	8.8%
Pack-years of smoking	13.6 (20.1)	14.9 (20.6)	14.9 (20.7)
Total activity in 1988 (MET-hrs/wk)	16.1 (17.9)	15.6 (16.7)	15.3 (17.7)
Ever oral contraceptive use	43.1%	40.5%	44.0%
Postmenopausal	83.3%	81.9%	82.7%
Current HT use	41.3%	42.5%	43.9%
Family history of diabetes	28.2%	30.9%	28.0%

Abbreviations: LTL, leukocyte telomere length; MET-hrs/wk, metabolic equivalent hours of activity per week; HT, postmenopausal hormone therapy.

a Values are means(SD) or percentages, and standardized to the age distribution of the study population at blood collection.

b Values may not add to 100% because of missing data.

c Value not age-adjusted.

After adjustment for age and case status, BMI-related variants near *GNPDA2* (rs10938397) and *RPL27A* (rs4929949), as well as one variant for T2D near *RBMS1*-*ITGB6* (rs7593730) were nominally associated (*P*≤0.05) with shorter LTL (Tables S1 and S2 in File S1). However, the number of associated variants did not exceed that expected by chance (*P* = 0.19 for BMI; *P* = 0.60 for T2D), and these variants became non-significant after False Discovery Rate correction for multiple testing.

For body mass, the mean genetic risk score was 29.2 and the standard deviation (SD) was 3.9; for T2D, the mean score was 38.4 and the SD was 4.0. As expected, positive associations among the genetic risk scores, body mass, and T2D were highly significant, suggesting these scores were reasonable proxies for BMI or T2D (Table 2). After adjustment for age and case status, each SD increase in the BMI genetic score was associated with a 0.62 kg/m^2^ increase in BMI (SE: 0.06; *P*<0.001), and each SD increase in the T2D score was associated with a 27% increased odds of T2D (95% CI: 1.13, 1.43; *P*<0.001). Moreover, the test statistics were >10 (the convention for identifying sufficiently strong genetic proxies for environmental exposures [53]) for associations between the scores and BMI (*F* = 29.0) or T2D (*χ^2^* = 12.7).

**Table 2:** Associations among body mass index, type 2 diabetes, and their respective genetic risk scores, Nurses' Health Study, 1989–1990.

		Quartiles of genetic risk score				
		1^st^	2^nd^	3^rd^	4^th^	*P* trend^b^
**Body mass index**						
*N*	–	973	1036	960	999	–
Score range (median)	–	16.0–26.4 (24.6)	26.5–29.2 (27.9)	29.3–31.8 (30.5)	31.9–42.9 (33.6)	–
LS mean kg/m^2^ (95% CI)^a^	–	23.6 (23.4, 23.9)	24.3 (24.0, 24.5)	24.7 (24.4, 25.0)	25.3 (25.0, 25.5)	<0.001
β (SE) per SD of score	0.62 (0.06)	–	–	–	–	–
**Type 2 diabetes**						
*N* (cases)	–	989 (60)	999 (64)	974 (78)	1006 (100)	–
Score range (median)	–	24.1–35.6 (33.7)	35.7–38.4 (37.0)	38.5–41.1 (39.6)	41.2–52.9 (43.0)	–
Odds ratio (95% CI)^a^	–	1.00 (ref)	1.02 (0.71, 1.48)	1.38 (0.97, 1.96)	1.70 (1.22, 2.38)	<0.001
OR (95% CI) per SD of score	1.27 (1.13, 1.43)	–	–	–	–	–

Abbreviations: LS mean, least squares mean; CI, confidence interval; SD, standard deviation; SE, standard error.

a Adjusted for age in years (continuous), case status (case, control).

b *P* values are 2-sided.

After combining the number of risk alleles across multiple risk variants, the scores for body mass and T2D were not associated with LTL (Table 3). For the BMI score, the least squares mean LTL (*z*-score) across quartiles of the genetic risk score were −0.005, −0.02, −0.02, and −0.04, respectively (*P*-trend = 0.52). For the T2D score, the least squares mean LTL (*z*-score) across quartiles of the genetic risk score were 0.02, −0.05, −0.07, and 0.01, respectively (*P*-trend = 0.69). Additional adjustment for BMI or T2D did not appreciably alter the findings. To assess whether the inclusion of cases influenced our findings, we repeated our analyses after restricting to women selected as controls (*N* = 2,222), as well as after excluding women selected as T2D cases (*N* = 310 excluded); we observed similar results to those from the main analyses (data not shown).

**Table 3:** Least squares mean telomere length (*z*-score) and 95% CI by genetic risk scores of common risk variants associated with higher body mass index or type 2 diabetes, Nurses' Health Study, 1989–1990.

		Quartiles of genetic risk score				
		1^st^	2^nd^	3^rd^	4^th^	*P* trend^b^
**Body mass index**						
*N*	–	973	1036	960	999	–
Score range (median)	–	16.0–26.4 (24.6)	26.5–29.2 (27.9)	29.3–31.8 (30.5)	31.9–42.9 (33.6)	–
LS mean LTL (95% CI)^a^	–	−0.005(−0.07, 0.06)	−0.02 (−0.08, 0.04)	−0.02 (−0.08, 0.04)	−0.04 (−0.11, 0.02)	0.52
β (SE) per SD of score	−0.01 (0.02)	–	–	–	–	–
**Type 2 diabetes**						
*N*	–	989	999	974	1006	–
Score range (median)	–	24.1–35.6 (33.7)	35.7–38.4 (37.0)	38.5–41.1 (39.6)	41.2–52.9 (43.0)	–
LS mean LTL (95% CI)^a^	–	0.02 (−0.04, 0.08)	−0.05 (−0.11, 0.01)	−0.07 (−0.13, −0.004)	0.01 (−0.05, 0.07)	0.69
β (SE) per SD of score	−0.006 (0.02)	–	–	–	–	–

Abbreviations: LS mean LTL, least squares mean leukocyte telomere length (*z*-score); CI, confidence interval; SD, standard deviation; SE, standard error.

a Adjusted for age in years (continuous), case status (case, control).

b *P* values are 2-sided.

For the T2D genetic risk score, results were consistent across unhealthy lifestyle pattern (*P*-interaction = 0.34). For the BMI score, however, estimates appeared stronger among women with an unhealthy lifestyle pattern compared to those with a healthy lifestyle (Figure S1 in File S1), although this difference was not statistically significant (*P*-interaction = 0.11).

Based on the correlations in our population between the genetic scores and BMI or T2D, as well as the correlations in the existing literature between these conditions and LTL, we calculated the statistical power in our study to detect an association between the scores and LTL (α = 0.05). Given that 2% of the variability in BMI was explained by variability in the genetic score, our data provided 80% power to detect an association between the score and LTL if at least 9% of the variability in LTL is explained by variability in BMI. Estimates of 1–18% have been reported among studies that identified an association [15], [16], [17], [54], [55], [56]. For T2D, 0.4% of the variability was explained by variability in the genetic score. Our data provided 80% power to detect an association between the score and LTL if at least 50% of the variability in LTL is explained by T2D status. Previous findings have been inconsistent, with reported estimates of <1–59% [18], [19], [20], [57], [58], [59]. These calculations suggest our data were underpowered to detect weak or modest effects; however, they provided sufficient power to detect previously reported associations of greater magnitude.

## Discussion

In this population of middle-aged and older women of European ancestry, we examined the joint effects of established BMI- or T2D-related risk variants on LTL. After combining data from multiple risk variants, women genetically predisposed to higher BMI or T2D did not possess shortened telomeres after adjustment for age and case status.

While our study is the first to examine the association between genetic predisposition to higher BMI or T2D and telomere length, many cross-sectional studies have directly examined the relation with these metabolic conditions. For BMI, some studies [14], [15], [16], [17], [55], [56], [60], [61], [62], including the present analysis, observed an inverse association with telomere length, while others reported no association [8], [21], [22], [63]. Similarly for T2D, most studies [12], [18], [19], [20], [57], [58], [59], [64], [65], including the present analysis, observed an inverse association despite a large variation in the strength of the relation. Although the reasons for these discrepancies are unclear, studies differed in sample size and population characteristics. In addition, these analyses had two important limitations. First, unmeasured confounders may have influenced findings. For example, several studies reported only unadjusted [22], [23], [58], [62] or age-adjusted results [12], [15], [57], [63], increasing the possibility of unmeasured confounding by factors such as age, smoking, or physical activity (i.e., potential predictors of higher BMI or T2D and telomere length). Second, cross-sectional analyses were unable to test the direction of the association between higher BMI or T2D and telomere shortening, although several studies hypothesized that telomere shortening may be a consequence of higher body mass or T2D due to elevated oxidative stress and inflammation [10], [11], [12], [13]. In the present analysis, we used the genetic risk scores as proxies for BMI and T2D exposure to test the causal role of these metabolic conditions in telomere biology [28]. As genetic variants are generally unaffected by environmental factors, our observations were minimally influenced by confounding. More importantly, telomere length is unlikely to affect genotypes, which enabled us to test whether higher BMI or T2D precede telomere shortening. Because neither genetic score was associated with LTL in the present analysis, we did not find evidence of a causal relation of strong magnitude, as findings from some studies suggested [17], [20], [55], [61], between higher BMI or T2D and LTL among middle-aged or older women of European ancestry.

Few prospective studies have examined whether higher BMI or T2D predict telomere shortening. Our findings were consistent with those of Farzaneh-Far *et* *al*. [66], who observed no association among 608 individuals between baseline BMI or T2D and subsequent telomere shortening over five years. O'Callaghan *et* *al*. [54], however, observed that men who lowered their BMI had increased telomere length over one year, although this study included only 54 men and requires replication in a larger study of women. Findings from recent studies have suggested that telomere length may also play a role in the pathogenesis of these metabolic conditions, which may account in part for previous cross-sectional associations. Njajou *et* *al*. [24] observed among 2,721 individuals that, unexpectedly, longer telomeres were associated with greater increases in BMI over seven years. In addition, Zee *et* *al*. [25] observed that genetic variants in telomere-related genes may predict risk of T2D among ∼22,000 women of European ancestry. Although requiring replication, these findings and those from the present analysis suggest relations among BMI, T2D, and telomere length are complex. Thus, although higher BMI or T2D may play a causal role of weak or modest magnitude in telomere shortening, these may not completely explain the observed cross-sectional associations.

Our null findings should be interpreted with caution given several limitations of our study. First, although the genetic risk scores captured the combined information from established genetic factors for higher BMI and T2D [26], [38], [67], [68], they accounted for only a small amount of the variability in these conditions – indicating that the influence of these scores on higher BMI or T2D is likely weak relative to the influence of other factors (e.g., environmental predictors, gene-environment interactions). Thus, when examining the null association with LTL it is important to consider that these scores do not capture comprehensively exposure to higher BMI or T2D. More specifically, they reflect a component of the genetic risk to these conditions and do not capture the non-genetic components, which may be relevant for LTL. Although this is an important limitation of our approach, large prospective studies of telomere length are often impractical as they require repeated measurements of LTL over time. Thus, using genetic scores as proxies for exposures to evaluate causal relations, with careful consideration of the limitations, provides a feasible alternative. In addition, future studies can use a similar approach by using telomere-related genetic variants as proxies to evaluate whether shortened telomeres predict higher BMI or risk of T2D, similar to Zee *et* *al*. [25].

Second, because the genetic scores were weak proxies of higher BMI and T2D, our data were underpowered to detect causal relations of weak or modest magnitude among BMI, T2D, and LTL. While our data provided sufficient power to detect large effects, the detection of weak associations using these scores requires large study populations that may only be available in meta-analyses (e.g., *N*>33,000 unrelated individuals if BMI or T2D explains 1% of the variability in LTL). Alternatively, future studies can potentially leverage the presence of gene-environment interactions to improve statistical power. For example, studies may be able to detect weak or modest effects on LTL by focusing on populations with unhealthier lifestyles, where genetic factors may better predict BMI or T2D [50], [51], [69]. While our data were underpowered to detect interactions, we observed that estimates between the BMI score and LTL appeared more pronounced among women with unhealthy lifestyle patterns expected to increase weight gain, supporting this possibility.

Third, because blood samples were stored on average for 19 years before LTL measurement, LTL degradation during storage is a possible concern. Because technicians were masked to participants' genotypes when measuring LTL, degradation would likely attenuate the results. However, years of sample storage was not correlated with telomere length in our population (*r*_s_ = −0.01, *p* = 0.64), and previous studies in the NHS have identified significant associations with telomere length [70], [71], [72], further supporting the validity of our telomere length measurement.

Lastly, our study population comprised women of European ancestry, which minimized population stratification. However, because telomere dynamics may differ among African Americans and Hispanics [7], [73], [74], our null findings may not be generalizable to women of other ethnicities.

In summary, we found that genetic predisposition to higher BMI or T2D, estimated by genetic risk scores, was not associated with shortened telomeres among middle-aged and older women of European ancestry. Although we cannot exclude weak or modest effects, our findings do not support a causal role of strong magnitude for these metabolic conditions in telomere dynamics. Future efforts to detect causal relations of weaker magnitude will likely require meta-analyses of existing and future genetic studies of telomere length, or large prospective studies with repeated telomere length measurements.

## Supporting Information

File S1 Supporting information file comprising Tables S1 and S2, as well as Figure S1. Figure S1 in File S1. Association between body mass index genetic score and leukocyte telomere length, by unhealthy lifestyle pattern. Figure shows change in leukocyte telomere length (*z*-score) and 95% confidence interval per additional 10 BMI-increasing risk alleles stratified by number of high-risk lifestyle practices, Nurses' Health Study, 1989–1990. Y-axis represents estimates of the β-coefficient adjusted for age in years (continuous) and case status (case, control). *P* value is 2-sided.
(DOCX)
