# Supplementary material for: Genetic Predisposition to Higher Body Mass Index or Type 2 Diabetes and Leukocyte Telomere Length in the Nurses' Health Study
Source: PLoS One. 2013 Feb 12;8(2):e52240. doi: 10.1371/journal.pone.0052240 (PMC3570546; doi:10.1371/journal.pone.0052240)
Supplement: File S1 — Supporting information file comprising Tables S1 and S2, as well as Figure S1. Figure S1 in File S1. Association between body mass index genetic score and leukocyte telomere length, by unhealthy lifestyle pattern. Figure shows change in leukocyte telomere length (z-score) and 95% confidence interval per additional 10 BMI-increasing risk alleles stratified by number of high-risk lifestyle practices, Nurses' Health Study, 1989–1990. Y-axis represents estimates of the β-coefficient adjusted for age in years (continuous) and case status (case, control). P value is 2-sided. (DOCX) [file pone.0052240.s001.docx]

**FILE S1**

Table S1. Associations between 32 risk variants of higher body mass index and mean telomere length (*z*-score), Nurses’ Health Study, 1989-1990

|  | **SNP** | **Gene** | **Chr** | **Risk** | **Risk** | **Per** | **SE** | ***P* ^b,c^** |
| --- | --- | --- | --- | --- | --- | --- | --- | --- |
|  |  | **region** |  | **allele** | **allele** | **allele** |  |  |
|  |  |  |  |  | **freq** | **β^a^** |  |  |
|  | rs1514175 | *TNNI3K* | 1 | A | 0.41 | -0.003 | 0.023 | 0.899 |
|  | rs1555543 | *PTBP2* | 1 | C | 0.59 | 0.028 | 0.023 | 0.231 |
|  | rs2815752 | *NEGR1* | 1 | A | 0.64 | -0.031 | 0.023 | 0.174 |
|  | rs543874 | *SEC16B* | 1 | G | 0.19 | 0.013 | 0.028 | 0.639 |
|  | rs2867125 | *TMEM18* | 2 | C | 0.82 | 0.004 | 0.029 | 0.891 |
|  | rs2890652 | *LRP1B* | 2 | C | 0.17 | 0.008 | 0.030 | 0.800 |
|  | rs713586 | *RBJ* | 2 | C | 0.48 | -0.011 | 0.022 | 0.618 |
|  | rs887912 | *FANCL* | 2 | T | 0.29 | -0.041 | 0.025 | 0.096 |
|  | rs13078807 | *CADM2* | 3 | G | 0.21 | 0.036 | 0.027 | 0.183 |
|  | rs9816226 | *ETV5* | 3 | T | 0.82 | -0.014 | 0.029 | 0.638 |
|  | rs10938397 | *GNPDA2* | 4 | G | 0.44 | -0.044 | 0.023 | 0.050 |
| § | rs13107325 | *SLC39A8* | 4 | T | 0.07 | -0.057 | 0.043 | 0.187 |
|  | rs2112347 | *FLJ35779* | 5 | T | 0.64 | -0.005 | 0.023 | 0.826 |
|  | rs4836133 | *ZNF608* | 5 | A | 0.49 | 0.036 | 0.022 | 0.113 |
|  | rs206936 | *NUDT3* | 6 | G | 0.20 | 0.026 | 0.028 | 0.359 |
|  | rs987237 | *TFAP2B* | 6 | G | 0.19 | -0.024 | 0.029 | 0.398 |
|  | rs10968576 | *LRRN6C* | 9 | G | 0.30 | -0.006 | 0.024 | 0.802 |
|  | rs10767664 | *BDNF* | 11 | A | 0.78 | 0.023 | 0.027 | 0.386 |
|  | rs3817334 | *MTCH2* | 11 | T | 0.42 | -0.031 | 0.023 | 0.168 |
|  | rs4929949 | *RPL27A* | 11 | C | 0.51 | -0.044 | 0.022 | 0.045 |
|  | rs7138803 | *FAIM2* | 12 | A | 0.38 | 0.040 | 0.023 | 0.083 |
|  | rs4771122 | *MTIF3* | 13 | G | 0.21 | -0.001 | 0.027 | 0.982 |
|  | rs10150332 | *NRXN3* | 14 | C | 0.22 | -0.014 | 0.027 | 0.611 |
|  | rs11847697 | *PRKD1* | 14 | T | 0.04 | -0.005 | 0.056 | 0.927 |
|  | rs2241423 | *MAP2K5* | 15 | G | 0.78 | -0.001 | 0.027 | 0.984 |
|  | rs12444979 | *GPRC5B* | 16 | C | 0.86 | 0.008 | 0.033 | 0.810 |
|  | rs1558902 | *FTO* | 16 | A | 0.41 | 0.002 | 0.023 | 0.942 |
|  | rs7359397 | *SH2B1* | 16 | T | 0.39 | -0.025 | 0.023 | 0.265 |
|  | rs571312 | *MC4R* | 18 | A | 0.24 | -0.001 | 0.026 | 0.980 |
| ‡ | rs2287019 | *QPCTL* | 19 | C | 0.82 | 0.017 | 0.030 | 0.575 |
|  | rs29941 | *KCTD15* | 19 | G | 0.68 | 0.003 | 0.024 | 0.886 |
| § | rs3810291 | *TMEM160* | 19 | A | 0.69 | 0.025 | 0.024 | 0.315 |

Abbreviations: SNP, single nucleotide polymorphism; Chr., chromosome; freq., frequency; SE, standard error

^a^Adjusted for age in years (continuous), case status (case, control)

^b^*P* values are 2-sided; calculated using the additive genetic model

^c^All SNPs nonsignificant after False Discovery Rate correction for multiple testing

§SNPs imputed with 0.6 < MACH Rsq < 0.8

‡SNPs imputed with MACH Rsq ≤ 0.6

Table S2. Associations between 36 risk variants of type 2 diabetes and mean telomere length (*z*-score), Nurses’ Health Study, 1989-1990

|  | **SNP** | **Gene** | **Chr** | **Risk** | **Risk** | **Per** | **SE** | ***P* ^b,c^** |
| --- | --- | --- | --- | --- | --- | --- | --- | --- |
|  |  | **region** |  | **allele** | **allele** | **allele** |  |  |
|  |  |  |  |  | **freq** | **β^a^** |  |  |
|  | rs10923931 | *NOTCH2* | 1 | T | 0.10 | 0.006 | 0.037 | 0.865 |
|  | rs340874 | *PROX1* | 1 | C | 0.55 | 0.020 | 0.023 | 0.372 |
|  | rs243021 | *BCL11A* | 2 | A | 0.46 | 0.043 | 0.022 | 0.057 |
|  | rs2943641 | *IRS1* | 2 | C | 0.63 | 0.006 | 0.023 | 0.796 |
|  | rs7578597 | *THADA* | 2 | T | 0.89 | -0.009 | 0.036 | 0.797 |
|  | rs7593730 | *RBMS1-ITGB6* | 2 | C | 0.78 | -0.057 | 0.027 | 0.034 |
|  | rs780094 | *GCKR* | 2 | C | 0.59 | -0.004 | 0.023 | 0.879 |
|  | rs11708067 | *ADCY5* | 3 | A | 0.78 | 0.024 | 0.027 | 0.367 |
|  | rs1801282 | *PPARG* | 3 | C | 0.88 | -0.009 | 0.035 | 0.784 |
|  | rs4402960 | *IGF2BP2* | 3 | T | 0.32 | 0.018 | 0.024 | 0.460 |
|  | rs4607103 | *ADAMTS9* | 3 | C | 0.75 | -0.021 | 0.026 | 0.417 |
|  | rs10010131 | *WFS1* | 4 | G | 0.60 | -0.032 | 0.023 | 0.168 |
| § | rs4457053 | *ZBED3* | 5 | G | 0.30 | -0.004 | 0.025 | 0.871 |
|  | rs10946398 | *CDKAL1* | 6 | C | 0.32 | 0.009 | 0.024 | 0.688 |
|  | rs2191349 | *DGKB-TMEM195* | 7 | T | 0.55 | -0.029 | 0.023 | 0.206 |
|  | rs4607517 | *GCK* | 7 | A | 0.17 | -0.001 | 0.030 | 0.986 |
|  | rs864745 | *JAZF1* | 7 | T | 0.49 | -0.026 | 0.022 | 0.244 |
| § | rs972283 | *KLF14* | 7 | G | 0.52 | -0.025 | 0.022 | 0.258 |
|  | rs13266634 | *SLC30A8* | 8 | C | 0.70 | -0.016 | 0.024 | 0.510 |
|  | rs896854 | *TP53INP1* | 8 | T | 0.49 | -0.027 | 0.022 | 0.228 |
|  | rs10811661 | *CDKN2A-B* | 9 | T | 0.83 | 0.037 | 0.030 | 0.218 |
|  | rs13292136 | *CHCHD9* | 9 | C | 0.93 | 0.038 | 0.045 | 0.394 |
|  | rs1111875 | *HHEX-IDE* | 10 | C | 0.59 | 0.001 | 0.023 | 0.958 |
|  | rs12779790 | *CDC123-CAMK1D* | 10 | G | 0.19 | 0.050 | 0.029 | 0.082 |
|  | rs7903146 | *TCF7L2* | 10 | T | 0.29 | 0.009 | 0.025 | 0.716 |
| § | rs10830963 | *MTNR1B* | 11 | G | 0.29 | 0.027 | 0.025 | 0.277 |
|  | rs1552224 | *CENTD2* | 11 | A | 0.86 | -0.036 | 0.032 | 0.260 |
|  | rs2237892 | *KCNQ1* | 11 | C | 0.94 | 0.030 | 0.048 | 0.529 |
| § | rs231362 | *KCNQ1* | 11 | G | 0.52 | 0.024 | 0.023 | 0.282 |
|  | rs5215 | *KCNJ11* | 11 | C | 0.36 | 0.002 | 0.023 | 0.926 |
|  | rs1531343 | *HMGA2* | 12 | C | 0.09 | 0.060 | 0.038 | 0.117 |
|  | rs7957197 | *HNF1A* | 12 | T | 0.80 | -0.006 | 0.028 | 0.823 |
|  | rs7961581 | *TSPAN8-LGR5* | 12 | C | 0.28 | -0.046 | 0.025 | 0.067 |
| § | rs11634397 | *ZFAND6* | 15 | G | 0.67 | -0.046 | 0.024 | 0.053 |
|  | rs8042680 | *PRC1* | 15 | A | 0.32 | -0.020 | 0.024 | 0.408 |
| ‡ | rs757210 | *HNF1B-TCF2* | 17 | T | 0.38 | -0.015 | 0.024 | 0.520 |

Abbreviations: SNP, single nucleotide polymorphism; Chr., chromosome; freq., frequency; SE, standard error

^a^Adjusted for age in years (continuous), case status (case, control)

^b^*P* values are 2-sided; calculated using the additive genetic model

^c^All SNPs nonsignificant after False Discovery Rate correction for multiple testing

§SNPs imputed with 0.6 < MACH Rsq < 0.8

‡SNPs imputed with MACH Rsq ≤ 0.6

Figure S1.

*P* interaction = 0.11
